# Correction: Time to revisit the endpoint dilution assay and to replace the TCID_50_ as a measure of a virus sample’s infection concentration

**DOI:** 10.1371/journal.pcbi.1010877

**Published:** 2023-01-31

**Authors:** Daniel Cresta, Donald C. Warren, Christian Quirouette, Amanda P. Smith, Lindey C. Lane, Amber M. Smith, Catherine A. A. Beauchemin

There is a semantic error in the description of the ratio of TCID_50_ to expected infections or SIN in the text, where the ratio is described as being 1 over its correct value.

Throughout the article, the number of infections that 1 TCID_50_ is expected to cause has been incorrectly reported as either being 1.44 infections in theory or 1.781 infections when estimated via Reed-Muench (RM) or Spearman-Karber (SK) method. It should be the inverse of those quantities such that 1 TCID_50_ is expected to cause 0.6931 infection in theory or 0.5615 infection when estimated via the RM or SK method.

All occurrences of the numbers -1/ln(50%) = 1.44 and e^ɣ^ = 1.781 in the text should be replaced by -ln(50%) = 0.6931 and e^-ɣ^ = 0.5615, respectively. There is a single exception to this on page 6 where the re-computed ratios are correctly expressed as “(RM/1.781)/SIN” and “(SK/1.781)/SIN”.

Additionally, in the Discussion, the statement:

“While, in theory, the intended MOI can be obtained by multiplying the TCID_50_ by 0.7 (or rather ln(2) = 0.693), one should instead multiply by 0.561 to account for the overestimation of the TCID_50_ by RM and SK.”

Should read:

“While, in theory, the intended MOI can be obtained by inoculating with 1.44 TCID_50_ (-1/ln(50%)) to infect one cell, in reality 1.781 TCID_50_ estimated via the RM or SK method is required to infect one cell, to account for the method’s overestimation of the TCID_50_.”

This semantic error was confined to the text of the article. None of the analysis, conclusions, publicly available code, or web calculator are affected.
