# Supplementary material for: Time to revisit the endpoint dilution assay and to replace the TCID50 as a measure of a virus sample’s infection concentration
Source: PLoS Comput Biol. 2021 Oct 18;17(10):e1009480. doi: 10.1371/journal.pcbi.1009480 (PMC8553042; doi:10.1371/journal.pcbi.1009480)
Supplement: Attachment — Submitted filename: response.pdf [file pcbi.1009480.s001.pdf]

Response to reviewers for PCOMPBIOL-D-21-00213

Time to revisit the endpoint dilution assay and to replace the $TCID_{50}$ as a measure of a virus sample's infection concentration

Daniel Cresta, Donald C. Warren, Christian Quirouette, Amanda P. Smith, Lindey C. Lane, Amber M. Smith, and Catherine A. A. Beauchemin

August 16, 2021

We would like to thank the reviewers for their helpful comments which enabled us to improve our manuscript. Below, we respond to the reviewers' comments, point-by-point, indicating where in the manuscript corrections were made to address the comments, where applicable.

Associate Editor

Comment 1. Your paper was reviewed by an expert on the statistics of estimating ID50s, and two experimental virologists with a strong quantitative research agenda. The two experimental reviewers saw great value in the approach you are proposing. All reviewers agree that the paper could be improved by shortening it, focusing on describing your method. The more general parts on the various benefits or drawbacks of plaque-forming and dilutions assays could be skipped, especially because the experimentalists among the reviewers did not fully agree with the material presented. We also believe that your paper would improve by comparing your method to more recent advances in the estimation of TCID50 as Reed and Munch is outdated (although still used in some circles). Reviewer 1 gives a few starting references for a more comprehensive comparison with existing methods.

Re focusing the manuscript We made significant cuts, primarily to the Introduction, as we removed general discussions of the pros/cons of plaque vs endpoint dilution assays. Specific changes are listed below in our response to Reviewer #2's Comments.

Re comparing our method to *"more recent advances in the estimation of the* $TCID_{50}$*"* we found this to be problematic for 3 key reasons:

- Reed-Muench is not only still used in some circles, together with Spearman-Kärber, these two estimation methods remain the standard, most commonly used methods for estimating the $TCID_{50}$ concentration of a virus sample from an endpoint dilution assay. As such, in this specific context, they remain "the methods to beat".
- More importantly, our aim is not to better estimate the $TCID_{50}$, but to get rid of it altogether in favour of the more useful and meaningful SIN. While the 50% animal infectious dose [refs offered by Reviewer #1] might be a helpful measure in animal studies, the 50% tissue culture infectious dose is an unhelpful measure in the context of in vitro viral infection experiments, e.g. it cannot directly be used to obtain a desired multiplicity of infection (MOI). In terms of estimating the SIN, there is no comparison to be made.
- Finally, as Reviewer #1 correctly points out, and as we acknowledge in our manuscript (now even more clearly, see our response to Comment 2), the use of Bayesian inference to estimate the 50% infectious dose (whether it be $TCID_{50}$ or the 50% animal infectious dose) is not novel nor unique to midSIN. As such, midSIN's estimate of the 50% infectious dose would perform identically to any other Bayesian inference-based implementation of an equivalent likelihood function, provided others' implementation is bug-free and typo-free. Beyond the Bayesian inference based

approaches, there is little value in comparing methods to other approaches, such as the logit or probit estimation methods, since those are rarely (if ever) used to estimate the $TCID_{50}$ of a virus sample.

Reviewer #1

Comment 2. The article uses Bayesian probabilities to calculate the posterior probability distribution of the effective concentration of infectious particles in a viral stock. It also implements the calculation on the web. The article is unsuitable for publication in its present form. The authors should announce the software (e.g., Google "journal bioinformatics software"), confining their explanation to a few pages at most. The article omits an adequate survey of even rudimentary references. The end of the review lists some references relevant to similar problems in animal trials, not necessarily for citation, but as a start for searching for appropriate citations.

It is important to keep in mind that references are often field-specific. For example, the 2 references offered by the Reviewer are both specific to the field of animal infectious dose, and aim to minimize the number of animals required to establish this measure. While we agree that the general concept, and some of the mathematics, is shared between viral $TCID_{50}$ in vitro and animal infectious dose, there are important differences. For example, it would seem that much of the estimation methods from this field of application use the beta-Poisson dose-response. It is meant to capture the high degree of variability in infection susceptibility between animals. This assumption is not part of the RM and SK estimation methods, and is beyond the scope and focus of our current work.

Our article's focus is on replacing the application of Reed-Muench and Spearman-Kärber in the context of estimating the $TCID_{50}$ of a virus sample. We have provided references within this specific context, which we expect our readers will be more familiar with, specifically references [1–3, 5, 9] herein (see References at the end of this document). Many of these references themselves have an extensive list of references to earlier works, and none of them referenced the 2 publications the Reviewer provided as examples. There was generally little to no overlap between the set of references between the 2 sets of literature. This supports our assertion that references are often field- or even subfield-specific, but does not suggest to us that these other authors failed to survey *"even rudimentary references"*.

From our perspective, the critical value of references is to properly place one's contribution in the context of the existing literature in their field: how is the work similar to past work, and what does it contribute that is new or unique. We believe that our references and manuscript achieve this. Below Equation (5) in our manuscript, we stated:

> *Note that this expression is largely equivalent to that obtained by Mistry et al. [5].*

In response to this Reviewer's comment, we have revised this statement **[lines 362]** to extend its scope as follows:

> *Note that this expression is largely equivalent to that obtained by Mistry et al. [5] in the context of estimating the* $TCID_{50}$ *of a virus sample, and by many others in the broader context of infection dose quantification [7, 8].*

In the Introduction, we stated:

> *Many have proposed replacements for the RM and SK calculations with some based on logit or probit transforms of the data [1–3] and others on statistical analysis of the ED assay output [3, 5]. Sadly, none of these improvements were widely adopted, possibly due to a lack of visibility of these publications, or the lack of widespread awareness of the limitations of the RM and SK methods.*

This acknowledges that we are not the first to raise these issues, that others have proposed improvements, and that indeed some specifically proposed a statistical analysis approach as we do. To specifically address this Reviewer's concerns, we revised this **[lines 33]** to:

> *Many have proposed replacements for the RM and SK calculations based on logit or probit transforms of the data [1–3] or on statistical analysis of the ED assay output [3, 5], with some implemented as website applications [4, 6]. Sadly, none of these improvements were widely adopted to improve estimates of the* TCID_{50}*, possibly due to a lack of visibility of these publications, or the lack of widespread awareness of the limitations of the RM and SK methods. None proposed replacing the* TCID_{50} *measurement unit, with a more meaningful measure.*

The application of Bayes theorem to improving estimates of the TCID_{50} is not the novel aspect of our work, nor do we suggest that it is. We see our present work as contributing the following novel or somewhat unique elements to the existing literature:

SIN We do not merely suggest a way to estimate the TCID_{50}. Rather, we propose that the TCID_{50} be replaced with a new measure to express the infectivity of a sample: Specific INfections (SIN). The new measure is biologically meaningful and directly experimentally useful, as it relates more directly to MOI. It is the number of infections the sample is most likely to cause per mL. To our knowledge, this is novel and needed.

Web-app Distributing our method not just as a freely available software (that people will not bother to install) but as a website-based application that one can readily and freely use without any installation, from any device (phone, tablet, computer), while not novel, is at least uncommon. Ease of use is essential to adoption, which is the biggest hurdle to discarding RM and SK. Both Mistry et al [5] and Spouge [7] do also offer their estimation method as website applications. This was not properly acknowledged in our references or manuscript before, but is now (see above).

With the revisions made (see above), we feel that we have better positioned our work within the context of the existing literature.

Comment 3. Most of the authors' explanation is unnecessary. The discussion of the relative merits of plaque-forming and dilutions assays, e.g., is irrelevant. Each type of assay has its merits and drawbacks, but the decision to use one or the other is subordinate to experimental means and ends. The article can therefore take the use of a dilution assay as dependent on ends, as a given. The article motivates itself with the Spearman-Karber and Reed-Muench methods. Although the methods still appear in the literature, they have been discredited for at least 30 years. The article's notation also obscures the simplicity of its ideas. Psychological experiments have shown that mathematical subscripts should be single letters, preferably with mnemonic value, because lengthy subscripts slow readers' comprehension. To appreciate the point, replace q[noinf] by q (without subscript) in all equations.

Comparisons of the plaque and endpoint dilution assays have been removed in response to Reviewer #2's comments (see below). This addresses the first part of this Reviewer's comment.

With regards to the comment that the Reed-Muench and Spearman-Kärber estimation methods have been discredited, we somewhat disagree with this opinion. It is true that several papers before ours have shown or discussed in various ways the key issues with these methods, and have proposed alternative estimations. However, Reed-Muench and Spearman-Kärber estimators remain the standard to estimate the TCID_{50} of a virus sample. Furthermore, all of these are still just methods to estimate the TCID_{50}, whereas we propose to replace the TCID_{50} with SIN, as a more biologically meaningful and experimentally useful unit of infectivity measurement for a virus sample. We have, however, better placed our work in the broader context of infection dose estimation beyond viral TCID_{50} (animal infectious dose, quantitative microbial risk assessment) in response to this Reviewer's concerns (see above).

With regards to notation: we submit that this is a matter of preference and have chosen to retain our original notation.

Comment 4. The Bayesian probability model motivating the article is routine. Physically, infection is modeled by a Poisson likelihood. The article then gives a lengthy physical justification of the model prior. A routine non-informative prior may be preferable, but in any case,

a Bayesian posterior should not be sensitive to the prior but depend mostly on the data. Any lengthy physical justification of the prior is therefore irrelevant.

With regards to the first sentence: the motivation of the manuscript is not the Bayesian probability model. Rather, it is to replace the TCID_{50} with SIN as a measure of a virus' infectivity. But indeed the estimation of both/either, in our manuscript, is done via application of Bayes' theorem. It is also correct that, in our estimation method, the infection probability can be — and is — approximated as an exponential function of the specific infection (SIN) concentration of the sample, i.e. a Poisson distribution (Eqn. (2) in our manuscript).

With regards to our discussion of the prior, we feel that it provides important information about our choice of prior, as we will now explain.

"a routine non-informative prior may be preferable" The idea that there is such a thing as an uninformative prior is not entirely sound. It is based on the assumption that there exists a key property of the posterior which the prior should not alter, e.g., the posterior's mode or variance should be equal to that of the likelihood function. Under such an assumption, the prior is said to be uninformative if it meets this (often arbitrary) objective. Instead, we feel that the prior should be chosen based on physical concerns, i.e. to accurately represent what is known about the parameter(s). When it comes to repeated observations of a binomial distribution (Bernoulli trials), the generally recommended prior is its conjugate pair, the Beta distribution, characterized by shape parameters α and β. Some view $\text{Beta}(\alpha = \beta = 1)$, which corresponds to a uniform distribution, as an uninformative prior in that it reflects our lack of information regarding the parameter: we know it is bounded but have no reason to prefer a particular value over another. This is the prior and the justification we use in our manuscript. Others argue for the Jeffreys prior, in this case $\text{Beta}(\alpha = \beta = \frac{1}{2})$, which corresponds to the arcsine distribution. Physically, an arcsine prior assumes that C_{inf} is more likely to be either very large or very small (near its extremum bounds), and less likely to take on values in between. Though this prior better preserves some features of the likelihood function, it does not accurately reflect prior physical knowledge. We found this document which provides an argument for why neither choices can be considered uninformative as per its author's definition of what uninformative should be (`http://urn.kb.se/resolve?urn=urn:nbn:se:uu:diva-150541`). Rather, the document mostly shows why the concept of an uninformative prior is not helpful since it depends on what one means by uninformative. This is why we feel it is important for authors who use Bayes' theorem to clearly motivate their choice of prior.

"posterior should not be sensitive to the prior" The prior appears directly in the expression for Bayes theorem and in our own expression, e.g. see Eqns. (4) and (5) in our manuscript. As such, it will always contribute to the posterior distribution, and the choice of prior will shape what that contribution will be. The prior's impact on the posterior can sometimes be lessened, i.e. its choice can become irrelevant, as more measurements are taken. In practice, however, it is not always the case, and it is dangerous to assume that it is. This is why it is important to show (Fig. 7 in our manuscript) that the choice of a prior uniform in C_{inf} versus uniform in $\log_{10}(C_{\text{inf}})$, does visibly shift the posterior distribution for our example ED plate (illustrated in Fig. 1), from $10^{6.21}$ SIN/mL to $10^{6.18}$ SIN/mL, respectively. This shift of 0.03 in the $\log_{10}(C_{\text{inf}})$ is less than the 68% credible interval of ~ 0.1 for this estimate. This shows that, at least in the case of this example, our choice of prior did not result in a statistically significantly different estimate for the most likely SIN concentration in the sample. But it did shift the posterior's mode and bounds, so it is not completely insensitive to the prior. This is why a demonstration of the manner in and the extent to which the choice of prior impacts one's posterior should always be presented in manuscripts making use of Bayes' theorem. This is especially true when a number of different priors could be considered physically reasonable.

For these reasons, we think that our discussion motivating our choice of prior and demonstrating its impact on the posterior distribution is important. The fact that it is confined to the Methods section also means that it does not impact the readability of our manuscript, but will improve interested researchers' ability to understand and reproduce our approach.

If the Reviewer or Editor(s) feel that the arguments provided above should be incorporated into our manuscript to answer similar concerns that might arise in the minds of potential future readers, we are open to incorporating them into the manuscript. Given the Reviewer's concern that this section is already too long, we thought it best not to do so.

Reviewer #2

Comment 5. The paper makes two arguments: (1) endpoint dilutions assays such as TCID50 are better than plaque assays, and (2) the midSIN method is better than things like Reed-Muench for computing titers from endpoint dilution assays. The second of these points is definitely true, and forms the strong basis for the content of this paper. However, I don't think the first point (superiority of endpoint dilution over plaque assay) is clearly established, nor do I think it's at all necessary for this paper. I say this as someone who personally prefers endpoint dilution assays (TCID50) to plaque assay. But some virologists prefer plaque assays for a variety of reasons, including liking to see the plaques, the additional information they get from examining plaque sizes, etc. If I hadn't read all the way through because I was a reviewer, I would have dismissed this paper after the first few paragraphs as an opinion piece arguing for TCID50 over plaque assays, and not paid attention to any of the rest. I strongly recommend the authors focus on what they clearly objectively demonstrate (that the midSIN method is better than alternatives for computing endpoint titers), and dispense with the more subjective arguments based on experimental factors that make them personally prefer endpoint assays to plaque assays.

We agree that in this work we do not show/demonstrate experimentally that the endpoint dilution assay is better than the plaque assay. As such, we agree that it is best to focus on the endpoint dilution assay, and on the need to replace the TCID_{50} with the SIN as a measure of a virus sample's infectivity.

We have revised our manuscript title and abstract to remove comparisons and arguments relating to the plaque/focus forming assay. We have shortened the Introduction (from 116 lines down to 64 lines) by removing any discussion of the limitations of plaque/focus forming assays. The only statements on the limitations of plaque/focus forming assays remaining in our manuscript is one sentence in Results **[lines 116]** and one in the Discussion **[lines 268]**. We believe those 2 statements are concise, important and factual.

Comment 6. I think the paper would benefit from a clearer "intuitive layman's explanation" of what exactly is wrong with the Reed-Muench formula compared to midSIN. Right now there is little explanation in main text, and then highly technical details in Methods but not good bridging of these.

The mathematical expressions behind the 2 methods, and in particular their shortcoming, is not particularly simple to express in words. We have attempted to do so by introducing Figure 4 and associated caption (see the revised manuscript). The new accompanying text at the start of the section titled *"Comparing **midSIN**'s performance to that of the RM and SK methods"* **[lines 148]** reads:

> *Figure 4 provides a graphical representation of how the RM and SK methods estimate the* TCID_{50} *concentration from an ED assay. Simply stated, the RM and SK methods use geometric arguments to estimate the sample dilution at which 50% of wells would be infected. While they are sometimes accurate (Fig. 4A,B), their simplicity often leads to biased estimates (Fig. 4C,D).*

We hope that this new figure provides an intuitive visual explanation for how these methods work and how they go wrong.

Comment 7. Although this is more a stylistic comment and one that is ultimately at the authors' discretion, I'd suggest that the paper will have more impact if it's more succinct, has less vague discussion of experiments and philosophical issues of titers, and really cuts more quickly to the heart of the issue which is that they have an improved way to calculate endpoint

titers, they have implemented a calculator, and that their method allows calculation of how experimental choices (like dilution factor, number of dilution series, etc) affect accuracy.

Indeed, by addressing this Reviewer's other comments, we have done exactly that.

Comment 8. The number of acronyms introduced just in the abstract (RM, SK, etc) becomes overwhelming and decreases readability. Maybe some of the less commonly used acronyms could be eliminated in favor of just writing out the full phrase?

From the Abstract and the Author summary, we have removed all abbreviations except for units (PFU, FFU, SIN, $TCID_{50}$).

In the rest of the manuscript, we have kept endpoint dilution (ED), Spearman-Kärber (SK), Reed-Muench (RM) since those are also used as short-hands in the figures and indeed in the graphical output of midSIN. We kept units (PFU, FFU, $TCID_{50}$, SIN, etc.) as these are these are not acronyms (like mL for milliliter). We kept multiplicity of infection (MOI) and confidence interval (CI) which are commonly used.

The only other one would be RSV for respiratory syncytial virus, but we felt it was used sufficiently frequently over a sufficiently constrained area of the manuscript that it would not cause confusion.

We expect that these changes will have fully addressed the Reviewer's concerns.

Comment 9. Lines 6–17: another limitation of counting virions under a microscope is that it does not distinguish physical from infectious particles. The same is true for qPCR. This is a really serious limitation, moreso even than cost, etc. In fact, I sort of wonder if this entire first paragraph is a little bit irrelevant to the question at hand, which is titrating infectious particles.

Indeed. These lines have been removed.

Comment 10. Lines 19–22: Again, this isn't quite true. They are certainly not easy to separate, but for instance with influenza there is some evidence that defective virions lacking genes sometimes have slightly different morphologies, etc—and can at least be partially separated by certain types of centrifugation. Again, like for lines 6–17, I sort of feel like the authors are spending a lot of time on not 100% accurate text that isn't even really relevant to their main point and finding, which is titrating infectious particles.

Lines 19–20 have been removed, but we have kept lines 21–22 (now **[lines 7–8]**). We felt it was important to explain the distinction between counting virions that are in principle infectious but might not go on to cause infection, and the number of infections caused, and to highlight the fact that infectivity assays actually measure infections caused rather than infectious virions. We believe this addressed the Reviewer's concern, but we are open to further changes if there are concerns with the lines that were kept.

Comment 11. Lines 68–71: The same limitation can apply to endpoint dilution (e.g., TCID50) assays, as the actual cell being used for the experiment doesn't always work for the endpoint dilution assay. For instance, people performing flu infections of human primary airway cells still titer the virus by TCID50 on MDCK cells as you can't do a TCID50 in human primary airway cells.

Indeed. These lines have been removed.

Reviewer #3

We broke up the 2 parts of Reviewer #3's comments [that were labelled 1) and 2)] into additional parts to provide a more targeted response to each aspect of the comments.

Comment 12. 1) There are many ways to quantify a virus sample and its important to consider what the assays measure. The authors aim at improving the estimation of an infection concentration meaning how many infections a virus sample could cause per unit volume. They compare plaque/focus forming assays with endpoint dilution methods. While the introduc-

tion gives a nice overview of these assays and highlights their limitations, in particular those of the plaque assay, it's not a fair comparison. The plaque or focus assays use an overlay medium to restrict infection spread to only the neighboring cells. Infected cells and their infected neighbors will then after some time and following some form of coloring be visible as a plaque/focus. The ED assay (TCID50) typically does not use overlay medium and relies on immunostaining or CPE to score infection. Plaque and ED assay thus have usually different readouts, replication or infection.

In response to Reviewer #2's comments (in particular, see Comment 5), we have significantly shortened our Introduction, and have removed comparisons of the plaque/focus forming assay to the endpoint dilution assay. We believe this addresses the concern raised here.

Comment 13. In the paper, the authors use hemagglutinating units to quantify the amount of released virus in their TCID50 assays. That is yet a different measure and might in include non-replicating particles.

We believe this comment refers to the endpoint dilution assays performed on influenza A virus samples, and reported in the section titled *"Comparing SIN to $TCID_{50}$ and PFU virus sample concentrations"*, with their corresponding experimental methodology described in the Methods section titled *"$TCID_{50}$ assay"*.

We performed a regular endpoint dilution assay, i.e. incubate MDCK cells with increasing dilutions of our virus samples as described in Methods section titled *"$TCID_{50}$ assay"*. After 3 d–4 d, the inoculum in each well will have either resulted in the infection of (nearly) all MDCK cells in the well, or none, depending on whether the diluted inoculum contained any infectious virions. It is only at that stage that hemagglutination was used, not to quantify the virus produced, but to score each well as either infected or not.

Previously, this was not clear in our Methods which read:

> *Supernatants were used to do a hemagglutination (HA) assay with chicken red blood cells. HA assays were performed and read by 'Researcher A' or 'Researcher B' on their respective experiments.*

We have revised the statements to improve clarity. It now reads [lines 473]:

> *Supernatants from each of the MDCK-containing wells were transferred to a matching well in a 96-well U-bottom plate in the same configuration, and mixed with chicken red blood cells (*30 min*, room temperature). This enabled us to score each of the original MDCK-containing wells as either positive or negative for infection, based on whether their supernatant caused hemagglutination. This was performed and read by 'Researcher A' or 'Researcher B' on their respective experiments.*

The hemagglutination was only used to score wells, in a manner that is equivalent to staining with crystal violet or trypan blue to visualize cell death (in the case of a lytic virus), or staining with virus antibodies to detect the presence of infected cells or virions in the supernatant. In this context, all these methods are equivalent: either **infection did not take place** and no significant cell infection and/or death occurred and no virus progeny was produced released into the supernatant and no hemagglutination occurred; or **infection took place** and extensive cell infection and/or death occurred and significant virus progeny was released into the supernatant which caused hemagglutination.

As for the inclusion of non-replicating particles: if the progeny virus particles released into the supernatant of an infection well (whether they be replicating or non-replicating) cause hemagglutination, it indicates that a productive infection took place in that well. In this context, even a supernatant containing mostly non-replicating progeny particles would still indicate that an infection took place in that well.

Comment 14. In my opinion is the proposed midSIN platform best suited to analyze traditional ED assays where infection is labelled with antibodies and can be analyzed with an automated reader. The manuscript (title, intro and discussion) is thus misleading in several places by saying that midSIN overcomes the limitations of a plaque assay.

With regards to the first part of this comment: midSIN is suited to analyze the output of any ED assay, irrespective of what endpoint is chosen to establish whether infection occurred in a well (cell death, antibody staining for infection/virus, hemagglutination by supernatant, etc.), or whether or not the endpoint readout is automated or done manually/visually by the researcher. As with the Reed-Muench and Spearman-Kärber estimators, the theory that midSIN relies on merely requires that the researcher be able to identify whether or not infection took place in each well. We do generally agree that automation/automated reader is preferable to human judgement (see our response to Comment 15), but it is not a requirement of the method.

With regards to the second part of the comment: This concern is similar to that raised in Reviewer #2's comments (in particular, see Comment 5), in response to which we have removed comparisons of the plaque/focus forming assay to the endpoint dilution assay.

Comment 15. 2) Another aspect that I think could be improved is the requirement of a threshold to score a well as positive. Can the analysis be performed using raw plate analyzer readings (fluorescence units per well)? That would be ideal and remove all personal bias from the analysis.

In principle, we agree that an automated scoring (by a machine) is preferable to human judgement. On the other hand, plate analyzers can be quite expensive, as are the consumable compounds they require, such as fluorescent antibodies, or antibodies loaded with compounds that can precipitate in the presence of another (colorimeter). In contrast, staining with crystal violet, trypan blue, etc. is an inexpensive and efficient way to identify the widespread cellular pathogenic effect of infection by a lytic virus, as are red blood cell to identify the presence of progeny virus released into the supernatant of a well infected with a hemagglutination-capable virus. Since the aim of the ED assay is merely to establish whether or not infection occurred, the scoring of a well as having been infected or not, even when done manually/visually, is unlikely to be ambiguous.

Proper consideration of this matter would require direct comparisons of machine- vs human-scoring for various endpoints and viruses. This would be necessary to establish whether or not the scoring (well is infected vs not infected) itself makes a significant contribution to the overall accuracy of the measure. It could be that its contribution is negligible in the face of other sources of error or bias, such as inter-experimental variability, virus sample dilution accuracy, etc.

For this reason, we now raise this interesting question in the manuscript's Discussion (next to last paragraph), as a possible direction to explore in future work.

References

[1] W. R. Bryan. Interpretation of host response in quantitative studies on animal viruses. *Ann. N. Y. Acad. Sci.*, 69(4):698–728, 16 December 1957. `doi:10.1111/j.1749-6632.1957.tb49710.x`.

[2] Z. Govindarajulu. *Statistical techniques in bioassay*, chapter 4. The Logit Approach, pages 35–90. Karger, Basel; New York, 2nd edition, 2001. `doi:10.1159/isbn.978-3-318-00617-9`.

[3] D. D. LaBarre and R. J. Lowy. Improvements in methods for calculating virus titer estimates from $TCID_{50}$ and plaque assays. *J. Virol. Methods*, 96(2):107–126, August 2001. `doi:10.1016/S0166-0934(01)00316-0`.

[4] B. A. Mistry. Website application associated with [5]. URL: `http://www.bhavenmistry.com/SMOI/`.

[5] B. A. Mistry, M. R. D'Orsogna, and T. Chou. The effects of statistical multiplicity of infection on virus quantification and infectivity assays. *Biophys. J.*, 114(12):2974–2985, 19 June 2018. `doi:10.1016/j.bpj.2018.05.005`.

[6] J. L. Spouge. Website application associated with [7]. URL: `https://www.ncbi.nlm.nih.gov/CBBresearch/Spouge/html_ncbi/html/id50/id50.cgi`.

[7] J. L. Spouge. Statistical analysis of sparse infection data and its implications for retroviral treatment trials in primates. *Proc. Natl. Acad. Sci. U.S.A.*, 89(16):7581–7585, 15 August 1992. URL: https://www.ncbi.nlm.nih.gov/CBBresearch/Spouge/html_ncbi/html/id50/id50.cgi, doi:10.1073/pnas.89.16.7581.

[8] M. H. Weir, J. Mitchell, W. Flynn, and J. M. Pope. Development of a microbial dose response visualization and modelling application for QMRA modelers and educators. *Environ. Model Softw.*, 88:74–83, February 2017. doi:10.1016/j.envsoft.2016.11.011.

[9] N. H. Wulff, M. Tzatzaris, and P. J. Young. Monte Carlo simulation of the Spearman-Kaerber TCID50. *J. Clin. Bioinforma.*, 2(1):5, 13 February 2012. doi:10.1186/2043-9113-2-5.
